# Predictors for intravenous immunoglobulin resistance and coronary artery lesions in Kawasaki disease

**DOI:** 10.1186/s12969-017-0149-1

**Published:** 2017-03-21

**Authors:** Tian Xie, Ying Wang, Songling Fu, Wei Wang, Chunhong Xie, Yiying Zhang, Fangqi Gong

**Affiliations:** Children’s Hospital, Zhejiang University School of Medicine, No.57, Zhugan Lane, Hangzhou, 310003 People’s Republic of China

**Keywords:** Kawasaki disease, Intravenous immunoglobulin, Coronary artery lesions

## Abstract

**Background:**

To assess the predictors for intravenous immunoglobulin (IVIG) resistance and coronary artery lesions (CALs) in Kawasaki disease (KD).

**Methods:**

A total of 560 KD patients were reviewed retrospectively, including 410 complete KD (cKD) and 150 incomplete KD (iKD) patients. The laboratory data were compared between the IVIG-resistant and IVIG-responsive groups, as well as between the coronary artery lesions (CALs+) and without coronary artery lesions (CALs-) groups.

**Results:**

In the cKD patients, C-reactive protein (CRP) levels had a sensitivity of 65.52% and a specificity of 62.7% for predicting IVIG-resistance at a cutoff point of >100 mg/L. When albumin <32 g/L, the sensitivity and specificity for predicting IVIG-resistance were 72 and 83.19%, respectively. N-terminal pro-brain natriuretic peptide (NT-proBNP) levels had a sensitivity of 73.91% and a specificity of 76.43% for predicting IVIG-resistance at a cutoff point of >1300 pg/ml. Interleukin-6 levels had a sensitivity of 76.19% and a specificity of 61.59% at a cutoff value of >45 pg/ml. Erythrocyte sedimentation rate (ESR) levels had a sensitivity of 53.26% and a specificity of 64.14% for predicting CALs at a cutoff point of >75 mm/h.

In the iKD patients, the sensitivity and specificity for predicting IVIG-resistance were 80 and 54.1% when hemoglobin <110 g/L. When proportion of neutrophils >70%, the sensitivity and specificity for predicting IVIG-resistance were 68 and 66.94%, respectively. ESR levels had a sensitivity of 70.83% and a specificity of 65.81% for predicting IVIG-resistance at a cutoff point of >80 mm/h. NT-proBNP levels had a sensitivity of 78.57% and a specificity of 56.67% for predicting IVIG-resistance at a cutoff point of >360 pg/ml. Interleukin-6 levels had a sensitivity of 70.59% and a specificity of 66.28% at a cutoff value of >25 pg/ml. Interleukin-10 levels had a sensitivity of 64.71% and a specificity of 74.42% for predicting IVIG-resistance at a cutoff value of >8 pg/ml. ESR levels had a sensitivity of 61.82% and a specificity of 65.12% for predicting CALs at a cutoff point of >75 mm/h.

**Conclusions:**

The white blood cell count, proportion of neutrophils, hemoglobin, CRP, ESR, albumin, NT-proBNP, interleukin-6 and 10 may be effective predictors for IVIG resistance and CALs in KD patients.

## Background

Kawasaki disease (KD) is an acute, self-limited, systemic vasculitis that predominantly affects children younger than five years, and is the leading cause of acquired heart disease in children [1]. The pathogenesis of KD still remains unknown although nearly fifty years have passed since the first report by Tomisaku Kawasaki in 1967 [2]. Although intravenous immunoglobulin (IVIG) combined with aspirin were commonly adopted as the most important therapy protocol which could effectively decrease the incidence of coronary artery lesions (CALs), the high incidence of IVIG-resistant and CALs were reported in recent years [3–7]. Therefore, it is very important to predict IVIG-resistance and CALs in patients with KD. Scoring systems for predicting unresponsiveness to IVIG therapy [8, 9] and risk factors of CALs [3, 10–12] have been extensively studied. However, there is no consensus on the risk factors of IVIG-resistance and CALs because of low sensitivity and specificity in those studies, which may be attributed to the limited parameters, especially laboratory findings. The aim of our study was to assess predictors for IVIG resistance and CALs in patients with Kawasaki disease.

## Methods

We retrospectively reviewed the medical records of patients diagnosed with KD at Children’s Hospital, Zhejiang University School of Medicine, from May 2010 to May 2014. A total of 560 KD patients, including 410 complete KD (cKD) and 150 incomplete KD (iKD) patients were enrolled in this study. The present study was reviewed and approved by the ethics committee of the same institution. Informed consent was obtained from each patient’s parent or guardian. All patients received IVIG at a dosage of 1 g/kg/day for 2 days and oral aspirin at 30–50 mg/kg/day. The dosage of aspirin was reduced to 3–5 mg/kg/day for 8 weeks at 3 day post-normal temperature. Laboratory findings were acquired at hospital admission and before IVIG administration, which included white blood cell count (WBC), proportion of neutrophils (N%), hemoglobin (HB), C-reactive protein (CRP), erythrocyte sedimentation rate (ESR), albumin (ALB), N-terminal pro-brain natriuretic peptide (NT-proBNP), interleukin-6 (IL-6) and IL-10.

### Definition of KD

Complete KD was diagnosed when subjects had at least five of the following six principal clinical signs: 1) fever persisting for five or more days; 2) bilateral conjunctival congestion; 3) changes to the lips and oral cavity; 4) polymorphous exanthema; 5) changes to peripheral extremities; and 6) acute nonpurulent cervical lymphadenopathy [13]. Incomplete KD was defined as having four or fewer principal signs, with or without cardiac lesions [14].

### Definition of IVIG-resistant and CALs

The IVIG-resistant was defined as those patients who remained febrile 48 h after administration of initial IVIG or recrudescent fever [15].

Echocardiography was used to assess CALs, which was performed before initial treatment and repeated at the point of 1 week, 2 weeks and 4 weeks after the initial treatment. Coronary artery was considered abnormal if the internal lumen diameter >2.5 mm in children <3 years of age, >3 mm in children 3–9 years of age, >3.5 mm in children 9–14 years of age; the internal diameter of a segment measuring ≥1.5 times that of an adjacent segment; and the lumen was clearly irregular [16].

### Statistical analysis

Normally distributed data were presented as mean ± SD and assessed by Student’s *t* test; continuous data of non-normally distributed were expressed as median (interquartile range) and analyzed by the rank-sum test. Categorical data were presented as frequency (percentage) and compared by Chi-square test as appropriate. Receiver operating characteristic (ROC) curves were applied to determine the optimal cutoff values of laboratory findings, and multivariable logistic regression analysis was performed to evaluate the role of each parameter. All statistical analyses were undertaken using IBM SPSS Statistics 20.0 software. *p* < 0.05 was considered to be statistically significant.

## Results

### The basic demographic characteristics (Table 1)

**Table 1:** The basic demographic characteristics

	complete KD	incomplete KD	*p*
M/F	247/163	103/47	NS
Age(months)*	25(13–47.25)	21.5(10.75–40.25)	NS
IVIG-resistant, *n* (%)	30(7.32%)	26(17.33%)	*p*<0.01
CALs, *n* (%)	95(23.17%)	58(38.67%)	*p*<0.01

*M/F* males/females, *NS* no significant difference, * Values are the median (interquartile range); *IVIG* intravenous immunoglobulin, *CALs* coronary artery lesions

A total of 560 KD patients were enrolled in this study, with 410 (73.2%) cKD patients and 150 (26.8%) iKD patients. The male to female ratio was 1.67:1 (350:210), with a ratio of cKD of 1.52:1 (247/163) and ratio of iKD of 2.19:1 (103/47). The patients with cKD had a median age of 25 months (interquartile range: 13–47.25 months); while the patients with iKD had a median age of 21.5 months (interquartile range: 10.75-40.25 months). No significant differences were observed in gender and age distribution between the cKD and iKD groups. Among these patients, 56 (10%) were unresponsive to the initial IVIG treatment, with 30 (7.32%) in the cKD group and 26 (17.33%) in the iKD group. During the follow-up, CALs were detected in 153 (27.32%) of the 560 patients with 95 (23.17%) in the cKD group and 58 (38.67%) in the iKD group. The incidences of IVIG-resistant and CALs in the iKD group were significant higher than those in the cKD group (17.33% *vs.* 7.32%, *P* < 0.01; 38.67% *vs.* 23.17%, *P* < 0.01; respectively). Additionally, the total incidence of CALs in the IVIG-resistant patients (27/56, 48.2%) was significantly higher than that in the IVIG-responsive patients (126/504, 25%).

### Predictors for IVIG-resistance

Table 2 showed that the levels of WBC, N%, CRP, ESR, IL-6 and IL-10 were significantly higher in the IVIG-resistant group compared to IVIG-responsive group both in cKD and iKD patients. The hemoglobin value was significantly lower in the IVIG-resistant group than in the IVIG-responsive group in iKD patients, but without significant difference in cKD patients. There were significant differences in the ALB levels between the IVIG-resistant and IVIG-responsive groups, and the ALB levels of IVIG-resistant patients were lower both in cKD and iKD patients. In cKD patients, the NT-proBNP level in the IVIG-resistant group was significantly higher than that in the IVIG-responsive group, but there was no significant difference in iKD patients.

**Table 2:** Comparisons of laboratory data between IVIG-resistant group and IVIG-responsive group both in cKD and iKD patients

	complete KD				*p*	incomplete KD				*p*
	[n] IVIG-resistant		[n] IVIG-responsive			[n] IVIG-resistant		[n]IVIG-responsive		
WBC (×10^9^/L)	[29]	18.69 ± 6.74	[380]	14.15 ± 5.67	0.001	[25]	20.17 ± 9.63	[148]	14.35 ± 5.94	0.000
*N* %	[30]	73.88 ± 14.36	[379]	63.62 ± 16.71	0.001	[25]	73.19 ± 16.43	[146]	59.97 ± 16.76	0.000
HB (g/L)	[30]	104.43 ± 12.48	[375]	108.03 ± 10.40	0.134	[25]	100.56 ± 12.00	[147]	109.53 ± 12.55	0.002
CRP (mg/L)	[29]	115.58 ± 48.87	[370]	82.08 ± 51.71	0.001	[25]	97.00 ± 50.49	[144]	66.70 ± 49.60	0.010
ESR (mm/h)	[29]	81.55 ± 31.15	[353]	65.81 ± 29.59	0.013	[24]	91.13 ± 35.27	[141]	63.22 ± 30.00	0.001
ALB (g/L)	[25]	30.33 ± 4.89	[339]	35.91 ± 4.29	0.000	[21]	32.67 ± 5.34	[131]	37.39 ± 4.31	0.001
NT-proBNP (pg/ml)	[23]	2192(607–4505)	[314]	512.5(210.5-1195.5)	0.000	[14]	775.(296.5–1780.75)	[104]	300.5(107.25–925)	0.094
IL-6 (pg/ml)	[21]	68.10(38.7–221.35)	[302]	29.55(10.45-93.25)	0.003	[17]	130.70(20.90–491.05)	[103]	11.90(5.07-50.58)	0.001
IL-10 (pg/ml)	[21]	14.40(5.25–60.05)	[302]	6.50(4.00–12.23)	0.006	[17]	12.10(4.8–45.6)	[103]	4.75(3.18–8.75)	0.001

Data were expressed as mean ± SD or median (interquartile range). n: number of patients, *WBC* white blood cell count, *N%* proportion of neutrophils, *HB* hemoglobin, *CRP* C-reactive protein, *ESR* erythrocyte sedimentation rate, *ALB* Albumin, *NT-proBNP* N-terminal pro-brain natriuretic peptide, *IL-6*: interleukin-6, *IL-10* interleukin-10

The laboratory parameters which had statistical differences were performed with multivariable logistic regression analysis to evaluate the relative risk of each parameter; and receiver operating characteristic (ROC) curves were used to calculate sensitivity and specificity (Tables 3 & 4). In cKD patients, a WBC cutoff value >13.18 × 10^9^/L had a sensitivity of 89.66% and a specificity of 46.32% for predicting IVIG-resistance; and a WBC cutoff value >16 × 10^9^/L yielded a sensitivity of 55.17% and a specificity of 69.47% for predicting IVIG-resistance. In iKD patients, when WBC >17.56 × 10^9^/L, the sensitivity and specificity for predicting IVIG-resistance were 64 and 77.24%, respectively. When WBC >20 × 10^9^/L, the sensitivity and specificity for predicting IVIG-resistance were 37.93 and 88.68% in cKD patients, and 48 and 84.55% in iKD patients, respectively.

**Table 3:** Multivariable logistic regression analysis of laboratory data for predicting IVIG-resistant in cKD patients

Cutoff	OR	*p*	Sensitivity, %	Specificity, %	PPV, %	NPV, %
	(95% CI)		(95% CI)	(95% CI)	(95% CI)	(95% CI)
WBC( × 10^9^ L)						
>13.18	7.803(2.327–26.161)	0.001	89.66(69.44–94.50)	46.32(41.36–51.34)	11.3(7.83–16.05)	98.32(95.19–99.43)
>16	3.002(1.412–6.385)	0.004	55.17(37.55–71.59)	69.47(64.67–73.89)	12.12(7.6–18.78)	95.31(92.14–97.24)
>20	4.524(2.017–10.145)	0.000	37.93(22.69–56)	88.68(85.1–91.49)	20.37(11.77–32.9)	94.9(92.13–96.77)
*N* (%)						
>70	2.341(1.096–5.001)	0.028	60(42.32–75.41)	60.42(55.42–65.22)	10.71(6.89–16.3)	95.02(91.5–97.13)
>80	4.561(2.117–9.83)	0.000	46.67(30.23–63.86)	83.91(79.87–87.26)	18.67(11.46–28.93)	95.2(92.36–97.03)
CRP(mg/L)						
>70	4.402(1.644–11.786)	0.003	82.76(65.45–92.4)	47.84(42.8–52.92)	11.06(7.55–15.93)	97.2(93.73–98.82)
>100	3.231(1.46–7.15)	0.004	65.52(47.35–80.06)	62.7(57.67–67.48)	12.1(7.89–18.13)	95.8(92.56–97.74)
ESR (mm/h)						
>50	2.441(0.908–6.559)	0.077	82.76(65.45–92.4)	33.71(28.98–38.79)	9.3(6.33–13.47)	95.9(90.91–98.27)
>100	2.438(1.055–5.634)	0.037	31.03(17.28–49.23)	84.42(80.27–87.83)	14.06(7.58–24.62)	93.7(90.49–95.89)
ALB (g/L)						
<32	11.719(4.689–29.288)	0.000	72(52.42–85.72)	83.19(78.84–86.79)	24(15.75–34.78)	97.5(95.09–98.82)
NT–proBNP(pg/ml)						
>1300	9.189(3.496–24.156)	0.000	73.91(53.53–87.45)	76.43(71.44–80.79)	18.68(12–27.9)	97.5(94.78–98.88)
IL–6 (pg/ml)						
>30	6.243(1.801–21.637)	0.004	85.71(65.36–95.02)	50.99(45.38–56.58)	10.84(6.97–16.49)	98.0(94.53–99.35)
>45	5.131(1.831–14.381)	0.002	76.19(54.91–89.37)	61.59(55.99–66.9)	12.12(7.6–18.78)	97.3(94.02–98.88)
>200	2.309(0.798–6.683)	0.123	23.81(10.63–45.09)	88.08(83.94–91.26)	12.2(5.32–25.54)	94.3(90.98–96.48)
IL–10 (pg/ml)						
>5	2.614(0.858–7.96)	0.091	80.95(60–92.33)	38.08(32.79–43.67)	8.33(5.27–12.94)	96.6(91.68–98.69)
>30	6.533(2.481–17.201)	0.001	38.1(20.75–59.12)	91.39(87.68–94.06)	23.53(12.44–40)	95.5(92.46–97.35)

*OR* odds ratio, *CI* confidence interval, *PPV* positive predictive value, *NPV*: negative predictive value, *WBC* white blood cell count, *N%* proportion of neutrophils, *CRP* C-reactive protein, *ESR* erythrocyte sedimentation rate, *ALB* Albumin, *NT-proBNP* N-terminal pro-brain natriuretic peptide, *IL-6* interleukin-6, *IL-10* interleukin-10

**Table 4:** Multivariable logistic regression analysis of laboratory data for predicting IVIG-resistant in iKD patients

Cutoff	OR	*p*	Sensitivity, %	Specificity, %	PPV, %	NPV, %
	(95% CI)		(95% CI)	(95% CI)	(95% CI)	(95% CI)
WBC ( × 10^9^/L)						
>17.56	6.032(2.406–15.12)	0.000	64(44.52–79.75)	77.24(69.07–83.75)	36.3(23.78–51.13)	91.35(84.37–95.38)
>20	5.053(2.004–12.736)	0.000	48(30.03–66.5)	84.55(77.13–89.88)	38.7(23.73–56.18)	88.8(81.91–93.39)
*N* (%)						
>70	4.303(1.712–10.817)	0.002	68(48.41–82.79)	66.94(58.15–74.69)	29.8(19.53–42.66)	91.0(83.25–95.37)
>80	8.721(3.142–24.21)	0.000	44(26.67–62.93)	91.74(85.45–95.45)	52.3(32.37–71.66)	88.8(82.08–93.21)
HB (g/L)						
<110	5.25(1.702–16.197)	0.004	80(60.87–91.14)	54.1(45.27–62.68)	26.3(17.73–37.18)	92.9(84.55–96.95)
CRP (mg/L)						
>70	3.804(1.476–9.803)	0.006	72(52.42–85.72)	59.66(50.68–68.04)	27.27(18–39.04)	91.0(82.62–95.58)
>100	3.776(1.548–9.209)	0.003	56(37.07–73.33)	74.79(66.3–81.73)	31.82(20–46.56)	89(81.37–93.75)
ESR (mm/h)						
>80	4.675(1.791–12.204)	0.002	70.83(50.83–85.09)	65.81(56.84–73.78)	29.8(19.53–42.66)	91.67(83.78–95.9)
>100	8.154(2.955–22.499)	0.000	45.83(27.89–64.93)	90.6(83.95–94.67)	50(30.72–69.28)	89.08(82.2–93.5)
ALB (g/L)						
<32	7.5(2.543–22.117)	0.000	42.86(24.47–63.45)	92.73(86.3–96.27)	52.9(30.96–73.83)	89.47(82.5–93.88)
NT–proBNP (pg/ml)						
>360	4.795(1.252–18.366)	0.022	78.57(52.41–92.43)	56.67(46.36–66.42)	22(12.75–35.24)	94.4(84.89–98.09)
>1300	2.000(0.553–7.231)	0.29	28.57(11.72–54.65)	83.33(74.31–89.63)	21.05(8.51–43.33)	88.2(79.68–93.48)
IL–6 (pg/ml)						
>15	9.474(2.04–43.995)	0.004	88.24(65.66–96.71)	55.81(45.29–65.84)	28.3(17.97–41.57)	96(86.54–98.9)
>25	4.717(1.516–14.676)	0.007	70.59(46.87–86.72)	66.28(55.78–75.38)	29.2(17.61–44.48)	91.9(82.47–96.51)
>120	15(4.242–53.042)	0.000	52.94(30.96–73.83)	93.02(85.6–96.76)	60(35.75–80.18)	90.9(83.07–95.32)
IL–10 (pg/ml)						
>8	5.333(1.764–16.125)	0.003	64.71(41.3–82.69)	74.42(64.29–82.46)	33.3(19.75–50.39)	91.4(82.53–96.01)
>30	8.542(2.008–36.328)	0.001	29.41(13.28–53.13)	95.35(88.64–98.18)	55.5(26.67–81.12)	87.23(79-92.54)

*OR* odds ratio, *CI* confidence interval, *PPV* positive predictive value, *NPV* negative predictive value, *WBC* white blood cell count, *N%* proportion of neutrophils, *HB*: hemoglobin, *CRP* C-reactive protein, *ESR* erythrocyte sedimentation rate, *ALB* Albumin, *NT-proBNP* N-terminal pro-brain natriuretic peptide, *IL-6* interleukin-6, *IL-10* interleukin-10

when HB <110 g/L, the sensitivity and specificity for predicting IVIG-resistance were 80 and 54.1%, respectively in iKD patients.

When N% >70%, the sensitivity and specificity for predicting IVIG-resistance were 60 and 60.42% in cKD patients, and 68 and 66.94% in iKD patients, respectively. When the N% cutoff value > 80% was applied, the sensitivity and specificity for predicting IVIG-resistance were 46.67 and 83.91% in cKD patients, 44 and 91.74% in iKD patients, respectively.

In cKD patients, CRP level had a sensitivity of 82.76% and a specificity of 47.84% for predicting IVIG-resistance at a cutoff point of >70 mg/L, and a sensitivity of 65.52% and a specificity of 62.7% for predicting IVIG-resistance at a cutoff point of >100 mg/L. Accordingly, the sensitivity and specificity for iKD patients were 72 and 59.66% at a cutoff point of >70 mg/L, 56 and 74.79% when CRP >100 mg/L.

In cKD patients, ESR level had a sensitivity of 82.76% and a specificity of 33.71% for predicting IVIG-resistance at a cutoff point of >50 mm/h, and had a sensitivity of 31.03% and a specificity of 84.42% at a cutoff point of >100 mm/h. In iKD patients, ESR levels had a sensitivity of 70.83% and a specificity of 65.81% for predicting IVIG-resistance at a cutoff point of >80 mm/h, and had a sensitivity of 45.83% and a specificity of 90.6% at a cutoff point of >100 mm/h.

When ALB <32 g/L, the sensitivity and specificity for predicting IVIG-resistance were 72 and 83.19% in cKD patients, 42.86% and 92.73% in iKD patients, respectively.

In cKD patients, NT-proBNP levels had a sensitivity of 73.91% and a specificity of 76.43% for predicting IVIG-resistantace a cutoff point of >1300 pg/ml. In iKD patients, NT-proBNP level had a sensitivity of 78.57% and a specificity of 56.67% for predicting IVIG-resistantat a cutoff point of >360 pg/ml, and had a sensitivity of 28.57% and a specificity of 83.33% at a cutoff point of >1300 pg/ml.

In cKD patients, IL-6 level had a sensitivity of 85.71% and a specificity of 50.99% for predicting IVIG-resistance at a cutoff value of >30 pg/ml, a sensitivity of 76.19% and a specificity of 61.59% at a cutoff value of >45 pg/ml, and a sensitivity of 23.81% and a specificity of 88.08% at a cutoff value of >200 pg/ml. In iKD patients, IL-6 level had a sensitivity of 88.24% and a specificity of 55.81% for predicting IVIG-resistance at a cutoff value of >15 pg/ml, and a sensitivity of 70.59% and a specificity of 66.28% at a cutoff value of >25 pg/ml, and a sensitivity of 52.94% and a specificity of 93.02% at a cutoff value of >120 pg/ml. In cKD patients, IL-10 level had a sensitivity of 80.95% and a specificity of 38.08% for predicting IVIG-resistance at a cutoff value of >5 pg/ml, and had a sensitivity of 38.1% and a specificity of 91.39% at a cutoff value of > 30 pg/ml. In iKD patients, IL-10 level had a sensitivity of 64.71% and a specificity of 74.42% for predicting IVIG-resistance at a cutoff value of >8 pg/ml, and had a sensitivity of 29.41% and a specificity of 95.35% at a cutoff value of >30 pg/ml.

### Predictor for CALs

Table 5 shows that the levels of WBC, ESR were significantly higher in patients with CALs than those in patients without CALs both in the cKD and iKD groups; whereas there were no statistical differences in the levels of N%, NT-proBNP, IL-6 and IL-10 between patients with CALs and those without CALs. The levels of HB and ALB were significantly lower in cKD patients with CALs than those without. CRP value was significantly higher in cKD patients with CALs than those without. In iKD patients, no significant differences were observed in the levels of HB, ALB and CRP between patients with CALs and those without.

**Table 5:** Comparisons of laboratory data between CALs + group and CALs- group both in cKD and iKD patients

	complete KD				*p*	incomplete KD				*p*
	[n] CALs+		[n] CALs-			[n] CALs+		[n] CALs-		
WBC (×10^9^/L)	[95]	16.61 ± 7.05	[314]	13.84 ± 5.31	0.000	[56]	17.31 ± 8.48	[92]	14.14 ± 5.67	0.007
*N* %	[95]	66.84 ± 15.41	[314]	63.63 ± 17.08	0.084	[55]	64.28 ± 17.42	[91]	61.00 ± 17.35	0.272
HB (g/L)	[94]	105.16 ± 11.96	[311]	108.55 ± 10.03	0.014	[55]	106.87 ± 12.90	[92]	108.68 ± 12.87	0.411
CRP (mg/L)	[89]	100.00 ± 53.47	[310]	80.07 ± 51.03	0.002	[53]	80.44 ± 51.63	[91]	67.02 ± 50.09	0.131
ESR (mm/h)	[92]	73.78 ± 32.29	[290]	64.85 ± 28.92	0.019	[55]	77.78 ± 33.60	[86]	61.70 ± 30.46	0.005
ALB (g/L)	[86]	33.48 ± 5.02	[278]	36.16 ± 4.21	0.000	[50]	35.81 ± 5.09	[81]	37.14 ± 4.56	0.134
NT-proBNP (pg/ml)	[76]	669.5(278.25–1583)	[261]	497(203–1374)	0.069	[39]	334(135–796)	[65]	303(90–1101.5)	0.869
IL-6 (pg/ml)	[71]	31.80(13.90–120.20)	[252]	30.45(10.86–94.66)	0.590	[37]	19.80(4.9–63.45)	[66]	13.35(6.70–56.33)	0.956
IL-10 (pg/ml)	[71]	6.30(3.7–13.4)	[252]	6.70(4.20–13.13)	0.748	[37]	5.80(3.35–10.80)	[66]	5.00(3.35–12.18)	0.921

Data were expressed as mean ± SD or median (interquartile range). n: number of patients, *WBC* white blood cell count, *N%* proportion of neutrophils, *HB* hemoglobin, *CRP* C-reactive protein, *ESR* erythrocyte sedimentation rate, *ALB* Albumin, *NT-proBNP* N-terminal pro-brain natriuretic peptide, *IL-6* interleukin-6, *IL-10* interleukin-10

Multivariable logistic regression analysis and receiver operating characteristic (ROC) curves were used to evaluate the relative risk, sensitivity and specificity of each parameter (Tables 6 and 7). In cKD patients, WBC level had a sensitivity of 62.11% and a specificity of 58.92% for predicting CALs at a cutoff value > 14.25 × 10^9^/L, and a sensitivity of 22.11% and a specificity of 89.49% at a cutoff value > 20 × 10^9^/L. In iKD patients, WBC level had a sensitivity of 60.71% and a specificity of 50% for predicting CALs at a cutoff value >14.25 × 10^9^/L, and a specificity of 35.71% and a specificity of 88.04% at a cutoff value > 20 × 10^9^/L. In cKD patients, when HB <110 g/L, the sensitivity and specificity for predicting CALs were 68.09 and 44.37%, when HB < 100 g/L, the sensitivity and specificity were 30.% and 82.96%, respectively. In cKD patients, CRP levels had a sensitivity of 94.38% and a specificity of 16.45% for predicting CALs at a cutoff point of > 25 mg/L, and had a sensitivity of 29.21% and a specificity of 82.26% at a cutoff point of > 140 mg/L. In cKD patients, ESR levels had a sensitivity of 53.26% and a specificity of 64.14% for predicting CALs at a cutoff point of > 75 mm/h. In iKD patients, ESR levels had a sensitivity of 61.82% and a specificity of 65.12% for predicting CALs at a cutoff point of >75 mm/h. When ALB<30 g/L, the sensitivity and specificity for predicting CALs in cKD patients were 23.26 and 90.65%, respectively.

**Table 6:** Multivariable logistic regression analysis of laboratory data for predicting CALs in cKD patients

Cutoff	OR	*p*	Sensitivity, %	Specificity, %	PPV, %	NPV, %
	(95% CI)		(95% CI)	(95% CI)	(95% CI)	(95% CI)
WBC ( × 10^9^/L)						
>14.25	2.35(1.467–3.766)	0.000	62.11(52.06–71.21)	58.92(53.4–64.22)	31.3(25.18–38.33)	83.71(78.27–88)
>20	2.416(1.321–4.421)	0.004	22.11(14.94–31.44)	89.49(85.61–92.42)	38.8(27.04–52.21)	79.1(74.63–83.06)
HB (g/L)						
<110	1.671(1.017–2.744)	0.043	68.09(58.11–76.64)	44.37(38.95–49.93)	27(21.75–32.99)	82.14(75.65–87.2)
<100	2.217(1.334–3.684)	0.002	30.85(22.42–40.79)	82.96(78.38–86.73)	35.3(25.89–46.16)	79.8(75.16–83.89)
CRP (mg/L)						
>25	3.308(1.278–8.561)	0.014	94.38(87.51–97.58)	16.45(12.74–20.98)	24.4(20.24–29.31)	91.0(80.74–96.13)
>140	1.913(1.113–3.29)	0.019	29.21(20.78–39.36)	82.26(77.62–86.11)	32.1(22.94–42.88)	80.19(75.46–84.2)
ESR (mm/h)						
>75	2.038(1.268–3.276)	0.003	53.26(43.14–63.12)	64.14(58.47–69.44)	32.0(25.15–39.78)	81.2(75.67–85.75)
ALB (g/L)						
<30	3.003(1.596–5.651)	0.001	23.26(15.59–33.21)	90.65(86.65–93.54)	43.4(30.21–57.75)	79.2(74.45–83.34)

*OR* odds ratio, *CI* confidence interval, *PPV* positive predictive value, *NPV* negative predictive value, *WBC* white blood cell count, *HB* hemoglobin, *CRP* C-reactive protein, *ESR* erythrocyte sedimentation rate, *ALB* Albumin

**Table 7:** Multivariable logistic regression analysis of laboratory data for predicting CALs in iKD patients

Cutoff	OR	*p*	Sensitivity, %	Specificity, %	PPV, %	NPV, %
	(95% CI)		(95% CI)	(95% CI)	(95% CI)	(95% CI)
WBC ( × 10^9^/L)						
>14.25	1.545(0.787–3.033)	0.206	60.71(47.63–72.42)	50(39.99–60.01)	42.5(32.26–53.43)	67.65(55.84–77.56)
>20	4.091(1.777–9.419)	0.001	35.71(24.46–48.81)	88.04(79.85–93.19)	64.5(46.95–78.88)	69.2(60.37–76.87)
HB (g/L)						
<110	1.676(0.845–3.323)	0.14	63.64(50.42–75.07)	32.61(23.89–42.72)	36.08(27.22–46)	60(46.18–72.39)
<100	1.231(0.582–2.603)	0.587	27.27(17.28–40.23)	76.09(66.44–83.64)	40.5(26.35–56.51)	63.6(54.33–72.02)
CRP (mg/L)						
>25	2.373(0.988–5.7)	0.053	84.91(72.95–92.15)	29.67(21.26–39.72)	41.2(32.49–50.67)	77.1(60.98–87.93)
>140	2.386(0.917–6.209)	0.075	20.75(12–33.46)	90.11(82.26–94.71)	55(34.21–74.18)	66.1(57.43–73.86)
ESR (mm/h)						
>75	3.022(1.498–6.097)	0.002	61.82(48.61–73.48)	65.12(54.59–74.35)	53.1(41.07–64.82)	72.7(61.88–81.42)
ALB (g/L)						
<30	2.474(0.74–8.273)	0.141	14(6.95–26.19)	93.83(86.35–97.33)	58.3(31.95–80.67)	63.8(54.93–71.94)

*OR* odds ratio, *CI* confidence interval, *PPV* positive predictive value, *NPV* negative predictive value, *WBC* white blood cell count, *HB* hemoglobin, *CRP* C–reactive protein, *ESR* erythrocyte sedimentation rate, *ALB* Albumin

## Discussion

Treatment with IVIG and aspirin significantly reduces the rate of coronary artery lesions. However, about 10% to 20% of children will have persistent or recrudescent fever after the initial IVIG therapy [4, 8, 17], and patients not responsive to IVIG therapy are at high risk of developing coronary artery lesions [7]. Therefore, Identification of the risk factors of IVIG-resistant and CALs is crucial. Kobayashi et al. [9] used demographic and laboratory characteristics, including age in months, day of illness at initial treatment, percentage of neutrophils, platelet count, and serum aspartate aminotransferase (AST), sodium, and CRP to generate a scoring model, which had a sensitivity of 86% and a specificity of 68% for predicting IVIG-resistance. Fukunishi et al. [18] had reported that serum levels of C-reactive protein, total bilirubin, lactate dehydrogenase, γ-glutamyltranspeptidase were significantly higher, and the hemoglobin value was significantly lower in the IVIG non-responsive group. In our research, the results of multivariable logistic regression analysis suggested that elevated WBC, N%, CRP, ESR, IL-6, IL-10 and low ALB level were independent risk factors for IVIG-resistance both in cKD and iKD patients. In the patients with iKD, HB<110 g/L can be diagnosed IVIG-resistant with OR = 5.25, while the difference of hemoglobin value between the IVIG-resistance and IVIG-responsive groups in cKD patients failed to reach statistical significance. Durongpisitkul et al. [17] found that low hemoglobin (<10 g/dl), high neutrophil count (>75%), high band count, and a low albumin were associated with unresponsive to the initial IVIG treatment. High levels of WBC, N%, CRP, ESR and anemia probably reflected the severity of ongoing inflammation in patients with KD.

In the present study, the levels of ALB in the IVIG-resistant group were significantly lower than the IVIG-responsive group both in cKD and iKD patients; there were very high sensitivity and specificity for predicting IVIG-resistant (72 and 83.19%) in cKD patients when ALB <32 g/L. As Terai et al. [19] revealed that the IVIG-resistant group had significantly severe hypoalbuminemia compared to the IVIG-responsive group. The vascular leakage was supposed as a key feature of KD pathophysiology, which led to hypoalbuminemia.

NT-proBNP is secreted and released by ventricular cardiomyocytes in response to myocardial wall stress and ischemia, which was widely used as an important biomarker of cardiovascular diseases [20]. A recent meta-analysis reported by Lin et al. [21] showed that NT-proBNP had high diagnostic value for identifying KD in patients with protracted undifferentiated febrile illness. Ye et al. [22] reported thatthe NT-proBNP levels in IVIG-non-responsive KD patients were significantly higher than IVIG-responsive KD patients (1023.5 *vs.* 2367.5). In our research, the NT-proBNP values in the IVIG-resistant group was significantly higher than in the IVIG-responsive group in cKD patients, but failed to reach significant difference in iKD patients. In cKD patients, when NT-proBNP>1300 pg/ml, the diagnosis sensitivity and specificity for predicting IVIG-resistance were 73.91 and 76.43%, respectively. Elevated NT-proBNP values may be associated with the vasculitis and myocarditis caused by KD.

Th1 and Th2 cytokines were considered helpful for predicting the disease prognosis and targeting treatment strategies in patients with KD as Wang et al. [15] reported previously. In our study, we found that the IL-6 and IL-10 values of the IVIG-resistant group were significantly higher than the IVIG-responsive group both in cKD and iKD patients, which was consistent with the study of Wang et al.

Previously, several studies have focused on risk factors for coronary artery lesions, particularly coronary artery aneurysms. Beiser et al. [10] developed instruments that incorporate baseline neutrophils and band counts, hemoglobin concentration, platelet count, and temperature on the day after infusion, to predict coronary artery abnormalities. However, the accuracy of the instrument was not consistently high in several validation studies, and the instrument may be inaccurate in patients who present after the tenth day of illness or who were diagnosed with iKD. In China, a recent study suggested that male gender, younger age, intravenous immunoglobulin dose, delayed administration, high platelet level and elevated ESR are predictive for CALs [6], but they defined CALs with the criteria of Japan Ministry of Health, which may be not suitable for Chinese population. In our study, the levels of WBC and ESR were significantly higher in patients with CALs compared to patients without CALs both in the cKD and iKD groups. In the cKD group, patients with high CRP, low levels of HB and ALB had a high risk for CALs.

The total incidence of CALs in IVIG-resistant patients was significantly higher than in patients responsive to IVIG in our research, which suggested that the formation of CALs was associated with IVIG-resistance. Interestingly, nearly half of the parameters in our study considered as effective predictors for IVIG-resistant but failed to reach statistical differences between patients with CALs and without CALs. It may be explained with the following points:1) The IVIG unresponsiveness may reflect only severity of ongoing inflammation, whereas development of CALs might be affected by both ongoing inflammation and hemodynamics [9]. 2) There are missing laboratory data in a proportion of patients. 3) In our study, criteria of CALs were different from other countries; in addition, patients had been diagnosed with CALs when echocardiogram was positive (including transient dilation) at any time during the follow-up, which might lead to misclassification and overestimate the prevalence of CALs. Therefore, prospective clinical large cohort trials are warranty.

## Conclusions

The levels of laboratory findings including WBC count, N%, HB, CRP, ESR, ALB, NT-proBNP, IL-6 and IL-10 can be used as predictors of IVIG-resistant andCALs. Especially, in cKD patients, when albumin<32 g/L, the sensitivity and specificity for predicting IVIG-resistance were72 and 83.19%, respectively. NT-proBNP level had a high sensitivity of 73.91% and a specificity of 76.43% for predicting IVIG-resistant at a cutoff pointof > 1300 pg/ml. In iKD patients, ESR levels had a sensitivity of 70.83% and a specificity of 65.81% for predicting IVIG-resistance at a cutoff pointof >80 mm/h. Interleukin-6 levels had a sensitivity of 70.59% and a specificity of 66.28% at a cutoff value of > 25 pg/ml. Interleukin-10 levels had a sensitivity of 64.71% and a specificity of 74.42% for predicting IVIG-resistance at a cutoff value of > 8 pg/ml. ESR levels had a sensitivity of 61.82% and a specificity of 65.12% for predicting CALs at a cutoff point of > 75 mm/h. Albumin and NT-proBNP were considered as the most effective predictors for IVIG resistance in the present study. Our study may be helpful for the treatment and long-term management of KD patients. Additional therapy and careful follow-up such as more frequent echocardiography should be considered in the patients with high risks for IVIG-resistant and CALs.

## Abbreviations

ALB: Albumin
CALs: Coronary artery lesions
CALs-: Patients without coronary artery lesions.
CALs+: Patients with coronary artery lesions
cKD: Complete kawasaki disease
CRP: C-reactive protein
ESR: Erythrocyte sedimentation rate
HB: Hemoglobin
iKD: Incomplete kawasaki disease
IL-10: Interleukin-10
IL-6: Interleukin-6
IVIG: Intravenous immunoglobulin
KD: Kawasaki disease
N%: Proportion of neutrophils
NT-proBNP: N-terminal pro-brain natriuretic peptide
WBC: White blood cell count
